# Correction: Bacterial communities in domestic washing machines associated with user-perceived odours

**DOI:** 10.1007/s00253-026-14005-7

**Published:** 2026-08-14

**Authors:** Sabasadat Seyednikkhoo, Mirko Weide, Markus Egert

**Affiliations:** 1 Faculty of Health, Medical and Life Sciences, Institute of Precision Medicine, Microbiology and Hygiene Group, Furtwangen University, Villingen-Schwenningen, Germany; 2 Microbiology, International R&D, Henkel Consumer Brands, Henkel AG & Co. KGaA, Düsseldorf, Germany

**Correction to: Applied Microbiology and Biotechnology**

10.1007/s00253-026-13972-1

In this article, Figure 5b was omitted; the complete Figure 5 should have appeared as shown below.

**Figure:**
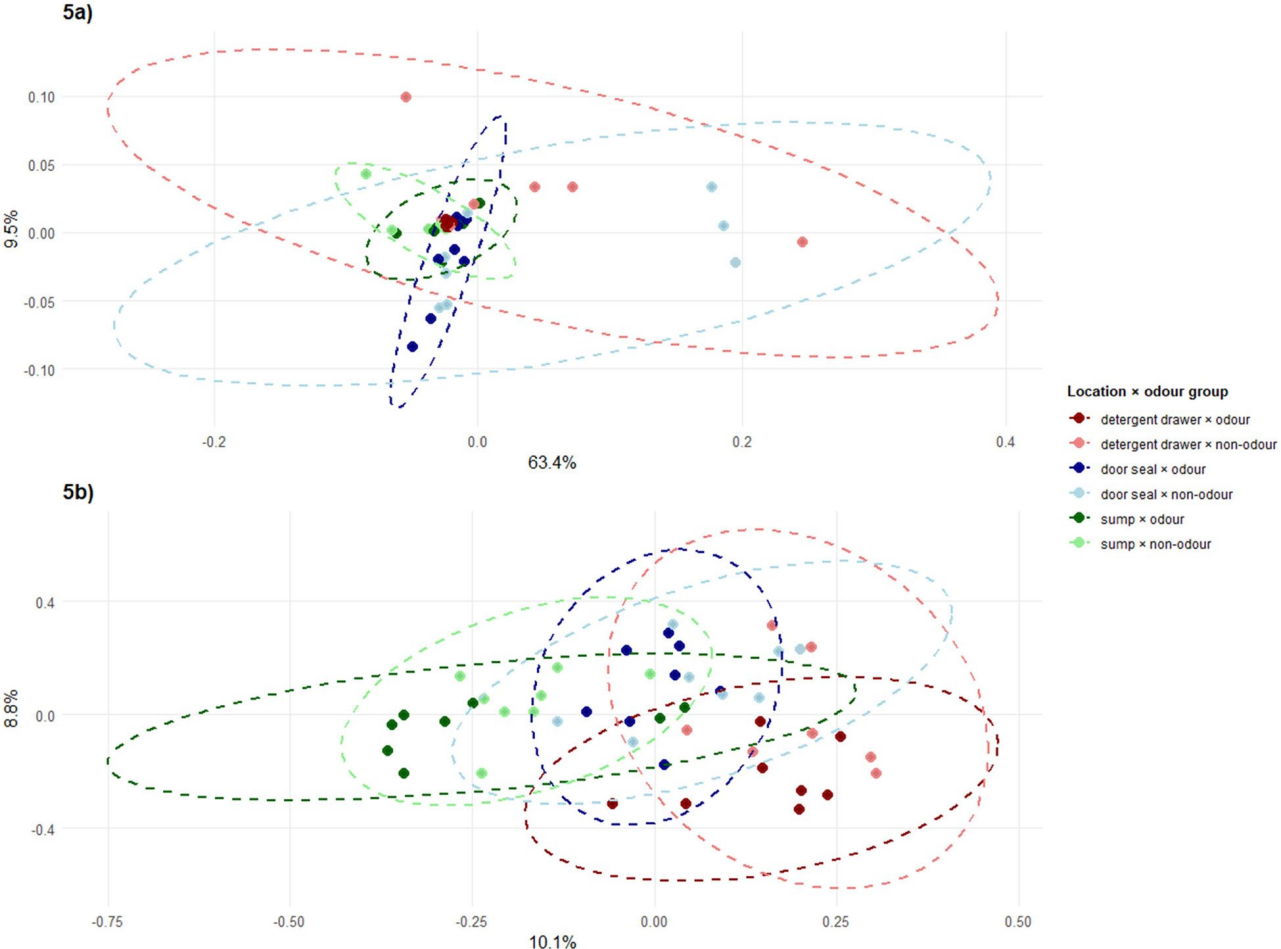

**Fig. 5** Principal coordinate analysis (PCoA) plot of weighted (**a**) and unweighted (**b**) Unifrac measures using the 16S rRNA gene sequencing data of 48 analysed washing machine samples. Colours indicate sampling site and smell group: door seal (blue range), detergent drawer (red range), sump (green range)

The original article has been corrected.

